# Ammonia leakage can underpin nitrogen-sharing among soil microorganisms

**DOI:** 10.1093/ismejo/wrae171

**Published:** 2024-09-05

**Authors:** Luke Richards, Kelsey Cremin, Mary Coates, Finley Vigor, Patrick Schäfer, Orkun S Soyer

**Affiliations:** School of Life Sciences, University of Warwick, Gibbet Hill Road, Coventry, CV4 7AL, United Kingdom; School of Life Sciences, University of Warwick, Gibbet Hill Road, Coventry, CV4 7AL, United Kingdom; School of Life Sciences, University of Warwick, Gibbet Hill Road, Coventry, CV4 7AL, United Kingdom; School of Life Sciences, University of Warwick, Gibbet Hill Road, Coventry, CV4 7AL, United Kingdom; Institute of Phytophathology, Justus-Liebig Universität, Heinrich-Buff-Ring 26-32 35392 Giessen, Germany; School of Life Sciences, University of Warwick, Gibbet Hill Road, Coventry, CV4 7AL, United Kingdom

**Keywords:** Microbial physiology, Microbial ecology, Soil nitrogen, Metabolic interactions, Mathematical modelling, Bacillus subtilis, Soil fungi, Serendipita indica, Microbial communities

## Abstract

Soil microbial communities host a large number of microbial species that support important ecological functions such as biogeochemical cycling and plant nutrition. The extent and stability of these functions are affected by inter-species interactions among soil microorganisms, yet the different mechanisms underpinning microbial interactions in the soil are not fully understood. Here, we study the extent of nutrient-based interactions among two model, plant-supporting soil microorganisms, the fungi *Serendipita indica*, and the bacteria *Bacillus subtilis*. We found that *S. indica* is unable to grow with nitrate - a common nitrogen source in the soil - but this inability could be rescued, and growth restored in the presence of *B. subtilis*. We demonstrate that this effect is due to *B. subtilis* utilising nitrate and releasing ammonia, which can be used by *S. indica*. We refer to this type of mechanism as ammonia mediated nitrogen sharing (N-sharing). Using a mathematical model, we demonstrated that the pH dependent equilibrium between ammonia (NH_3_) and ammonium (NH^+^_4_) results in an inherent cellular leakiness, and that reduced amonnium uptake or assimilation rates could result in higher levels of leaked ammonia. In line with this model, a mutant *B. subtilis* – devoid of ammonia uptake - showed higher *S. indica* growth support in nitrate media. These findings highlight that ammonia based N-sharing can be a previously under-appreciated mechanism underpinning interaction among soil microorganisms and could be influenced by microbial or abiotic alteration of pH in microenvironments.

## Introduction

The vast array of soil microorganisms and their interactions, the “soil microbial community”, confer a myriad of functions in soils. These functions underpin the contribution of soil community to biogeochemical cycles [1–7]. The latter contribution is mediated through microbial processing of nutrients needed by the plant, suppression of plant pathogens, enhanced stress tolerance (reviewed by [8]), and by modulation of plant hormone signalling [9, 10]. There is therefore a requirement to better understand soil microbial communities and the microbial interactions within, so to preserve and possibly increase crop/ soil fertility. Several studies have shown that abiotic environmental inputs influence soil and plant microbiome assembly [11–13] and the topic had been reviewed [7]. At the community level, nutrient availability has been shown to strongly influence community diversity and it is suggested that high nutrient availability promotes the proliferation of few competitive species and low nutrient availability promotes diversity [14]. External nutrient supply, as well as micro-scale nutrient gradients, are therefore important factors in shifting the balance of metabolic microbial interactions, as shown in experiments with model systems [14–19]. The environmental influences on community composition are likely mediated through direct effects on specific inter-microbial interactions, however, specific interactions among soil microorganisms are often not well-characterised. Generally speaking, interactions between microorganisms can take many forms from competitive to cooperative and can be mediated by a range of different mechanisms [20, 21]. Among these, metabolic interactions through cross-feeding and auxotrophy are shown to be wide-spread in many different environments, including the soil [15, 22–24]. Auxotrophic interactions occur when one organism loses ability to synthesize a growth-essential compound, and instead receives this from another organism [25].

Among specific soil microorganisms, previous work has highlighted mutual growth promotion between the fungus *Mortierella elongata* and bacterium *Burkholderia* BT03, mediated in-part by fungal organic acids [24]. An auxotrophic interaction has also previously been identified, via vitamin B1 (thiamine), between the bacterium *Bacillus subtilis* and the fungus *Serendipit indica* [15]. This specific interaction is notable because *S. indica* is an important soil fungi believed to promote plant growth and stress tolerance across a range of plant species [26–28]. The inability of *S. indica* to produce thiamine means that its beneficial activity towards plants is dependent on nutrient input from other soil organisms such as *B. subtilis* or the plants themselves. In addition to its inability to produce thiamine, *S. indica* has been shown previously to have impaired growth in media with nitrate as the only N-source, and lacks genes coding for nitrate transporters and, nitrate and nitrite reductases [29]. Considering that nitrate is a major nitrogen source in global soils and agricultural practices [11, 30–33], this raises a question about why fungi such as *S. indica* would lose their ability to assimilate such an abundant nitrogen source and how they sustain themselves in nitrate-dominant environments. One possible answer to these questions could be that alternative nitrogen sources, that are easier to utilise, readily co-existing with nitrate, in nitrate-rich environments due to activities of nitrate utilising bacteria.

Here, we have explored this hypothesis using the *B. subtilis* – *S. indica* pair as a model bacteria – fungi interaction system. We reconfirmed the incapability of *S. indica* to use nitrate as a sole nitrogen source and demonstrated that this incapacity is ameliorated by the presence of *B. subtilis*. We showed that this effect is mainly due to ammonia, which we found to be released into the media by *B. subtilis*. This finding is supported by a mathematical model, showing that ammonia can be readily leaked by cells due to its pH dependent equilibrium with ammonium and the high permeability of the latter through cell membranes. Thus, our results show that inevitable leakage of ammonia can act as a nitrogen sharing mechanism among soil microorganisms. This highlights the possible importance of incidental leakage of membrane-permeable metabolites in the development of auxotrophic interactions and the maintenance of soil microbial community stability.

## Materials and methods

### Growth media

Here we use modified ATS media [34]: 70 *μ*M H_3_BO_3_, 14 *μ*M MnCl_2_, 0.5 *μ*M CuSO_4_, 1 *μ*M ZnSO4, 0.2 *μ*M NaMoO_4_, 10 *μ*M NaCl, 0.01 *μ*M CoCl_2_, 2.5 mM KPO_4_ (2.3 mM KH_2_PO_4_ and 0.2 mM K_2_HPO_4_ for pH 5.8), 3 mM MgSO_4_, 3 mM CaCl_2_, 50 *μ*M Fe-EDTA, 100 mM glucose, 500 nM thiamine. N-sources were 10 mM KNO_3_, 5 mM (NH_4_)_2_SO_4_ or 5 mM glutamine. Where needed 2% agarose was added. Modified BG11+ media composition was as follows 1.5 g/L NaNo_3_, 0.04 g/L K_2_HPO_4_·3H_2_O, 0.02 g/L Na_2_CO_3_, 6 mg/L FeCl_3_·6H_2_O, 3 mg/L H_3_BO_3_, 2 mg/L MnCl_2_·4H_2_O, 0.39 mg/L Na_2_MoO_4_·2H_2_O, 0.22 mg/L ZnSO_4_·7H_2_O, 0.02 mg/L biotin, 0.02 mg/L folic acid, 0.1 mg/L pyridoxinehydrochloride, 0.05 mg/L thiamine hydrochloride, 0.05 mg/L riboflavin, 0.05 mg/L nicotinic acid, 0.05 mg/L D-calcium pantothenate, 0.05 mg/L para-aminobenzoic acid, 0.001 mg/L cabalamin, 0.05 mg/L lipoic acid, and 1% glucose.

### Microbial preparation

*S. indica* stocks (−80°C 500000 spores/mL in 0.02% tween_20_) were germinated on CM-agar plates (20 g/L glucose, 6 g/L NaNO_3_, 2 g/L peptone, 1 g/L casein hydrolysate, 1 g/L yeast extract, 1.52 g/L KH_2_PO_4_, 502 mg/L MgSO_4_·7H_2_O, 502 mg/L KCl, 6 mg/L MnCl_2_·4H_2_O, 2.65 mg/L ZnSO_4_·H_2_O, 1.5 mg/L H_3_BO_3_, 0.75 mg/L KI, 0.13 mg/L CuSO_4_·5H_2_O, 2.4 ng/L Na_2_MO_4_·2H_2_O, 15 g/L agar) and matured for 4 weeks. Agar plugs were transferred to fresh CM-agar plates and matured for minimum 8 weeks. Spores were harvested by washing with 0.02% tween_20_ solution and adjusting to 500 000 spores/mL using a Fuchs-Rosenthal haemocytometer. All bacterial strains: *Pseudomonas composti* (two strains) and *Allorhizobium rhizophilum* from [35] and *B. subtilis* strains NCIB3610, 168 and 168∆amtB were maintained at −80°C in 25% glycerol. 168∆amtB was generated by [36]. Glycerol scrapes were streaked on LB-agar (10 g/L Peptone, 5 g/L Yeast extract, 10 g/L NaCl, 1.5% Agar) plates and incubated at 30°C overnight. Single colonies from these plates were inoculated into 10 mL LB-broth for overnight cultures. After overnight growth the cells were spun at 12000 x g for 1 min, washed twice with 100 mM NaCl and adjusted to the desired optical density (OD_600_ = 0.5 unless otherwise stated) for use as inoculum.

### Plate inoculation of S.Indica and B. Subtilis experiments

For plating, 2 mL of 2% agarose ATS media in 3.5 cm Petri dishes was used as described above and N-sources were added at the concentrations indicated. To inoculate S. indica, 5 *μ*L of *S. indica* spore suspension was placed centrally onto plates for mono-culture experiments, plates were sealed with parafilm and allowed to grow for 3 weeks in the dark in static 30°C incubator. For co-culture experiments, the *S. indica* inoculum was slightly offset to one side to allow for 2 cm separation between organisms. These plates were placed in static 30°C incubator for 2 days prior to *B. subtilis* inoculation. After 2 days plates were opened and allowed to dry for around 1 hr in sterile conditions. For co-culture plates, 1 *μ*L of *B. subtilis* inoculum or 1 *μ*L 100 mM NaCl was placed on the opposite side of the plate. Plates were wrapped in parafilm and kept in the 30°C static incubator until imaging.

### Preparation of B. Subtilis supernatant

To create supernatant, 200 mL ATS media with 10 mM KNO_3_ was added to sterile 500 mL conical flasks and 1 mL of *B. subtilis* inoculum (prepared as described above) was added and cultures were placed in 30°C with 170 rpm shaking. For initial experiments considering a range of *B. subtilis* OD_600_ supernatants, cultures were sampled at approximately 24, 28, 32, and 52 hrs into the experiment. For later experiments, cultures were sampled at 28 hrs and the exact OD_600_ measured. The cultures were centrifuged at x3200g for 10mins followed by vacuum filter sterilisation of the supernatant through Corning PES filters 0.22 *μ*m for use in later experiments and analysis.

### S. Indica liquid culture experiments

*S. indica* liquid cultures were grown in 250 mL conical flasks filled to a final volume of 100 mL ATS with the indicated N-sources, *B. subtilis* supernatant and 100 *μ*L spore inoculum. These were placed in shaking 30°C incubator 170 rpm for 1 week before sampling. On sampling cultures were passed through Miracloth (Merck-Millipore, Burlington, MA, USA) to filter mycelia from the growth supernatant. Supernatant was collected for metabolite analysis and mycelia were scraped from the Miracloth surface and placed in 2 mL Eppendorf tubes to dry for weight measurements. Tubes and samples were dried at 70°C for 1–2 days and then returned to room temperature for around 24 hours before weighing to equilibrate to ambient humidity. Weights were measured using a Sartorius Secura 124–15 balance by measuring the tubes plus fungal material, then removing the fungal material and weighing only the tubes and calculating the difference.

### Cross species ammonium quantification

Bacterial inocula were prepared as described above to a density of OD_600_ = 1 in 0.9% saline. To initiate cultures, 100 *μ*L of inoculum was added to 100 mL BG11+ media (listed above) in a 250 ml conical flask. These cultures were incubated shaking at 30°C and sampled twice daily. OD measurements were taken regularly until OD *>* 0.5 was reached. Supernatant was collected by centrifugation and filter sterilisation. Ammonium was quantified by using the Supelco Spectroquant ammonium test kit and absorbance measured at 700 nm using CLARIOstar BMG Labtech plate reader.

### Fluorescence pH quantification

Fluorescence standard curve was constructed by preparing KPO_4_ buffer at four different pH values by combining different ratios of 1 M KH_2_PO_4_:K_2_HPO_4_ as follows: pH 4, 100:0 (adjusted down to pH 4 with 0.1 M HCl), pH 5.8 91.5:8.5, pH 6.8 50.3:49.7, pH 8 6:94. Propidium iodide (PI), for normalisation, and the pH sensitive 2′,7’-Bis-(2-Carboxyethyl)-5-(and-6)-Carboxyfluorescein (BCECF) (Invitrogen, Waltham, MA, USA) were added to these at a final concentration of 100 *μ*M and 10 *μ*M respectively. Solutions in 3.5 cm Petri dishes were imaged with bright field illumination with 0.5 ms exposure, HcRED1 filter set 41 043 (Chroma) exposure (100 ms) and EGFP filter set 41 018 (Chroma) exposure (100 ms). Light source was pE-300white (CoolLED). Standard curve for pH quantification is performed (Fig. S1A).

### Nitrate, ammonium and amino acid quantification

Quantification of free amino acids and ammonium, plus total protein content (amino acid content post acid hydrolysis) was conducted externally by Genaxxon (Ulm, Germany) using HPLC (high performance liquid chromatography) LC3000 with post-column ninhydrin derivitisation at 125°C. For detection of protein-derived amino acids, samples were hydrolysed, prior to HPLC separation, in 6 N HCl at 110°C for 60 hours. For detection of nitrate ions we used the Dionex (Sunnyvale, CA, USA) ICS-5000^+^ ion-chomotography system. We used an anionic column with KOH eluent. 2.5 *μ*L of sample (filtered through 0.22 *μ*m syringe filter) was injected and run at a flow rate of 0.38 mL/min for 30 minutes in a constant gradient as follows: 0 mins 1.5 mM KOH, 8 mins 1.5 mM KOH, 18 mins 15 mM KOH, 23 mins 24 mM KOH, 24 mins 60 mM KOH, 30 mins 60 mM KOH. Nitrate was detected at an elution time of around 12.8 minutes using a conductivity detector. Standard curve for quantification is performed (Fig. S1B).

### Mathematical modelling

We used a modified version of the model developed by [37] to consider the dynamics of ammonia and ammonium between the interior and exterior of a cell growing in a nitrate-only and given pH environment. We defined differential equations for the external (Equation 1) and internal (Equation 2) ammonium concentrations. 


(1) \begin{equation*} dN{H}_4\_\mathit{\operatorname{ext}}/ dt= kN- diff- NH4\_\mathit{\operatorname{ext}}\cdotp k1 \end{equation*}

(2) \begin{equation*} dN{H}_4\_\mathit{\operatorname{int}}/ dt= kN\_ in+ diff+N{H}_4\_\mathit{\operatorname{ext}}\cdotp k1-N{H}_4\_ in\cdotp kbm \end{equation*}

(3) \begin{equation*} diff=P\_ VA\left(k\_\mathit{\operatorname{ext}}\cdotp NH4\_\mathit{\operatorname{ext}}-k\_ in\cdotp NH4\_ in\right) \end{equation*}

Parameters *kN* and *kN_in* represent the external influx (from cells into the external environment) and internal influx (from nitrate reduction) of ammonium. Parameter *diff* represents diffusion into or out of the cell from the exterior environment and is given by Equation 3 where *P_V_A_* is the rate of ammonia diffusion from the cell and *k_in* and *k_ext* are the internal and external dissociation constants respectively for the ammonium-ammonia equilibrium at the pH of those compartments. The rates *k1* and *kbm* represent the rates of ammonium import into the cell and ammonium incorporation into biomass respectively. For the parameter *kN_in*, we inferred an amount of ammonium per hour that must be generated through nitrate reduction. We considered in the experiments, that there was a reduction of approximately 2.5 mM nitrate over the course of the 28 hrs growth (Fig. S2). Thereby indicating at least 90 *μ*M per hour (2500/28) of internal ammonium production at the population level.

We solved differential equations for change in external ammonium concentration (Equation 1) and internal ammonium concentration (Equation 2) until simulations reached steady state. We check steady state has been reached by verifying that the external ammonium concentration is not changing by more than 10^−5^ within experimental timeframes. Endpoint measurements were then used to generate surface plots. Python code for this analysis is available at: https://github.com/lukeZrich/RichardsNsharing2024.

### Plate reader assessment of bacterial growth

ATS broth was used for plate reader growth experiments with the following modifications. KPO_4_ buffer composition was altered to increase the pH by increasing the ratio of K_2_HPO_4_ to KH_2_PO_4_ such that the final media contained 1.3 mM KH_2_PO_4_ and 1.2 mM K_2_HPO_4_ for pH 6.8. To combat precipitation, we reduced the concentration of MgSO_4_ and CaCl_2_ by ten fold for 0.3 mM each. *B. subtilis* inocula were prepared as described above and 1 *μ*L used to inculate 200 *μ*L of media per well. Cultures were grown in a plate reader (CLARIOstar BMG Labtech) for 3 days at 30°C, 200 rpm and OD_600_ measurements taken every 30 minutes.

### BLAST searching of nitrogen assimilation genes homology

Protein gene accession as described in Data file S1 were used to perform protein–protein BLAST against the *S. indica* and *S. vermifera* genetic information stored in the NCBI (TaxID 1 109 443 and 109 899, respectively). Default settings were used to run Blastp [38] protein–protein Blast.

## Results

### *S. Indica* lacks the ability to assimilate nitrate

**Figure 1:**
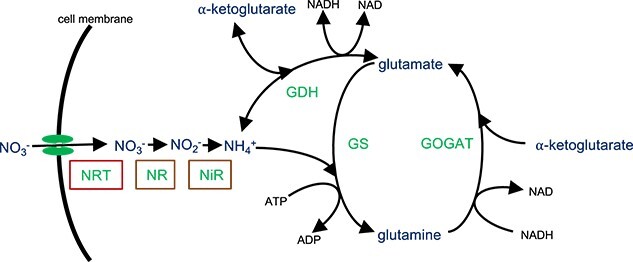
General pathway of nitrogen assimilation. Boxed enzymes are those not found in *S. Indica.* Nitrate can be taken up by active transport, via nitrate transporters (*NTR*), and reduced to nitrite and then ammonium by nitrate reductases (*NR*) and nitrite reductases (*NiR*). Ammonium can then be assimilated by two enzyme-facilitated reactions: Glutamate dehydrogenase (*GDH*) catalyzes the NADH-dependent reversible reaction of *α*-ketoglutarate with ammonium, resulting in glutamate, and glutamine synthase (*GS*) catalyzes the reaction of ammonium with glutamate, resulting in glutamine. A third enzyme, glutamate synthase (*GOGAT*), catalyzes the re-generation of glutamate by a reaction that combines glutamine and *α*-ketoglutarate.

**Figure 2:**
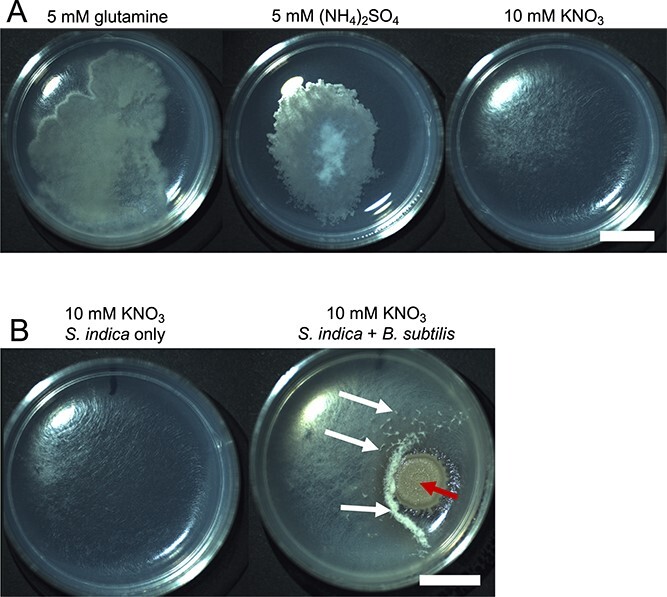
A) *S. Indica* growth after 42 days on ATS media supplemented with the indicated Nsource. B) *S. Indica* growth after 42 days in isolation or in the presence of *B. Subtilis* on ATS media supplemented with 10 mM KNO_3_. *B. Subtilis* Inoculum was added 2 days after *S. Indica* inoculation. On co-culture plates, large *B. Subtilis* colonies are visible on the right, indicated with a dark, left facing (red) arrow. In co-culture the mat of *S. Indica* mycelia appears generally more dense but is also accompanied by” fluffy” protrusions from the media, indicated with light, right facing (white) arrows. The two sets of images shown on the left and right panels are from replicate experiments. Scale bar is 1 cm and applies to all panels.

Both bacteria and fungi, generally, use the same metabolic pathways for the assimilation of nitrate into amino acids (Fig. 1) [39, 40]. Using the pBLAST tool [38], and genes from *Bacillus subtilis* as a reference, we searched both the *S. indica* and *Serendipita vermifera* NCBI sequence repositories for *GDH* (Glutamate dehydrogenase), *GS* (Glutamine synthase), and *GOGAT* (glutamate synthase) homologs, as well as for *NTR* (nitrate transporters), NR (nitrate reductase), and *NiR* (nitrite reductase) (see *Methods*). For *GDH*, *GS*, and *GOGAT* we found likely homologs in both fungal species with a *>* 25% sequence identity match over *>*89% of the query sequence length. For *NTR*, *NR*, and *NiR* possible homologs were found in the *S. vermifera* genome with *>*26% sequence identity match over *>*42% of the query sequence length but no hits were identified in *S. indica* for *NTR* and those identified for *NiR* and *NR* had lower identity and length than that quoted for *S. vermifera*. Additionally, the annotations for those hits do not match *NR*s and *NiR*s (Data file S1). To explore this further, we used the *Ogatea angusta* formerly *Hansenula polymorpha* yeast species’ nitrate assimilation genes *OaYNT1* nitrate transporter, *OaYNR1* nitrate reductase, and *OaYNI1* nitrite reductase [41] to search the *S. indica* and *S. vermifera* genomes for homologous genes. This represents an example of a well-characterised nitrate-assimilating fungus [42]. Likely homologs were identified for all three genes in the *S. vermifera* genome but only the nitrate reductase (*OaYNR1*) produced hits in the *S. indica* genome (Data file S1). These had low sequence similarity over only the latter portion of the query sequence where the NADH binding domain is located in other fungal *NR*s [43]. These results, and in particular the lack of *NTR*s, suggest that *S. indica* will be unable to use nitrate as the sole nitrogen source. To experimentally test the capability of *S. indica* to use nitrate, we grew *S. indica* spores on ATS media containing different nitrogen sources: nitrate, ammonium, and glutamine (see *Methods*). *S. indica* displayed extremely reduced growth on media containing nitrate when compared with ammonium and glutamine (Figs. 2A and S3A).

### S. Indica growth in nitrate media is significantly enhanced in the presence of B. Subtilis

Given that *S. indica* can readily use ammonia and glutamine, we hypothesised that soil bacteria may be able to provide *S. indica* with these alternative nitrogen sources in a nitrate-dominated environment. To investigate this, *S. indica* was grown on ATS-nitrate plates with and without the addition of *B. subtilis*. *B. subtilis* improves the growth of *S. indica* on agar plates (Figs. 2B and S3B), leading to the hypothesis that *B. subtilis* is providing some exuded nitrogen compound capable of diffusing across the media and being taken up by *S. indica*.

**Figure 3:**
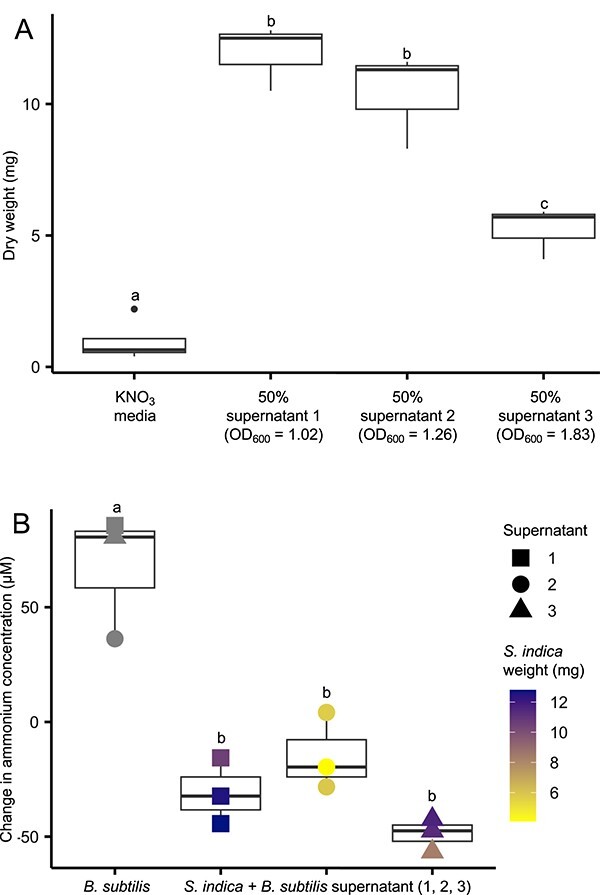
A) Dry weight of *S.Indica* growth after 1 week of liquid culture in ATS media supplemented with 10 mM KNO_3_ or a 50:50 mixture of this media and supernatant of a *B. Subtilis* culture grown in the same media. Harvesting OD_600_ for this supernatant is indicated on the x-axis. B) Difference between end-point and starting NH^+^_4_ concentration, as measured by HPLC, in *S. Indica* and *B. Subtilis* cultures in conditions indicated on the x-axis. Where applicable *S. Indica* dry weight (from a) for each individual point is indicated with a colour scale. For both figures mid point indicates median, edges of boxes indicate lower (LQ) and upper quartiles (UQ) and ends of whiskers indicate maxima and minima excluding outliers, defined as points outside the bounds LQ/UQ −/+ 1.5·IQR (inter-quartile range). Different letters above boxes indicate a significant difference (TukeyHSD *P <* 0.05). Plot point shapes indicate the OD_600_ of the *B. Subtilis* culture at the time of supernatant harvest (from a): Diamond - NA, square - 1.02, circle - 1.83, triangle - 1.26.

To further investigate nitrogen sharing between *B. subtilis* and *S. indica*, we assessed growth of *S. indica* in liquid culture in the presence of *B. subtilis* supernatant. Trial experiments indicated that, qualitatively, *B. subtilis* supernatant promoted the growth of *S. indica* in liquid culture (Fig. S4A). Nitrate and ammonium quantification of the *B. subtilis* supernatant samples used in these experiments shows consumption of nitrate as OD increases and a production of ammonium (Fig. S4). At higher optical densities growth promotion was reduced, possibly because of a re-consumption of ammonium by *B. subtilis* at high bacterial cell densities (Fig. S4). To re-confirm this growth-promotion result quantitatively, we designed experiments focusing on *B. subtilis* supernatant collected at an OD below 2 (Fig. S5 and *Methods*). The *B. subtilis* supernatant addition greatly increased the dry mass of *S. indica* grown in liquid culture over 1 week (Figs. 3A and S6), supporting the hypothesis that exudates from *B. subtilis* are used as a nitrogen source by *S. indica*. To further confirm this observation, we repeated the supernatant supplementation experiment (Fig. 3A) and again found a significant growth promotion of *S. indica* with *B. subtilis supernatant* (Fig. S7). We also quantified the nitrate in supernatants from these experiments (see *Methods*) and were able to show a clear reduction in the amount of nitrate in *B. subtilis* supernatants which was not seen in the supernatants of *S. indica* (Fig. S2). Thus, *B. subtilis* growth results in nitrate consumption and a release of a nitrogen source into supernatant that can be used by *S. indica*.

### *S. Indica* uses ammonia released by *B. Subtilis* to grow in the nitrate-only media

To identify the compounds in the supernatant of nitrate-grown *B. subtilis* that *S. indica* could use as N-source, we analysed supernatant samples by HPLC. We could not detect any free amino acids in any of the supernatant samples other than those collected at OD greater than 4 (Data file S1). We also tested supernatant samples for the presence of protein-derived amino acids by subjecting them to acid hydrolysis prior to HPLC quantification. The sum of protein-derived amino acids was not significantly different between samples and generally the amounts detected were low (Fig. S2). High amounts of ammonia and taurine were detected in all of these hydrolysis-treated samples, including media only controls, indicating that hydrolysis can result in production of these compounds from media components.

The lack of any significant excreted or protein-derived amino acids in supernatant of nitrate-grown *B. subtilis*, led us to hypothesise that the effect of the supernatant on *S. indica* could be due to ammonium. It has been noted that ammonia (NH_3_) exists in equilibrium with ammonium (NH^+^_4)_ and that the former can readily leak in and out of the cell due to its high permeability to the membrane [37]; making it an ideal candidate for such a cross-feeding interaction. If leakage of ammonia is an unavoidable process, this would also explain the observation of ammonium in *S. indica* only samples (Fig. S8), as some of the nitrogen stored in spores would be lost through ammonia leakage during its utilisation. Leakage of ammonia would also complicate the assessment of ammonium usage by *S. indica* when grown in *B. subtilis* supernatant, as consumption and leakage would occur simultaneously. In order to correct for this, we subtracted the mean ammonium concentration observed in *S. indica*-only cultures (Fig. S8). We subsequently calculate the change in ammonium concentration over the course of the experiment and compare these to the change in ammonium concentration in *B. subtilis* only cultures (Fig. 3). We find that in cultures where *S. indica* is placed in media with *B. subtilis* supernatant there is clear consumption of ammonium. This effect is correlated with *S. indica* growth (Fig. 3), supporting the conclusion that *S. indica* is using this leaked ammonium to support growth.

To further verify that ammonia levels found in *B. subtilis* supernatant can promote *S. indica* growth to the levels seen, we supplemented *S. indica* with the same levels of ammonium found in *B. subtilis* supernatant. We found that a supplementation of ammonium, equivalent to 37.5 μM nitrogen can significantly enhance *S. indica* growth in nitrate media (Fig. S9). Further confirming ammonia as mediator of the *B. subtilis* and *S. indica* interaction in these experiments, the lowest ammonium producing *B. subtilis* culture - one with the highest OD_600_ - produced the smallest growth benefit to *S. indica* (Fig. 3). These findings strongly support the notion that the growth promotion effect of *B. subtilis* supernatant on *S. indica* in the nitrate-media is due to ammonium transferred between these organisms.

The leakage and sharing of ammonia is likely to be a universal phenomenon. To investigate this idea, we grew *B. subtilis* and three additional microbial strains (*Allorhizobium rhizophilum* and two strains of *Pseudomonas composti* [35]) in growth media known to support their growth (BG11) with nitrate as the sole nitrogen source. We found that all bacterial strains produced micromolar amounts of ammonium (Fig. S10).

### Mathematical modelling of ammonia assimilation highlights the role of environmental pH on ammonia leakage and microbial interactions

**Figure 4:**
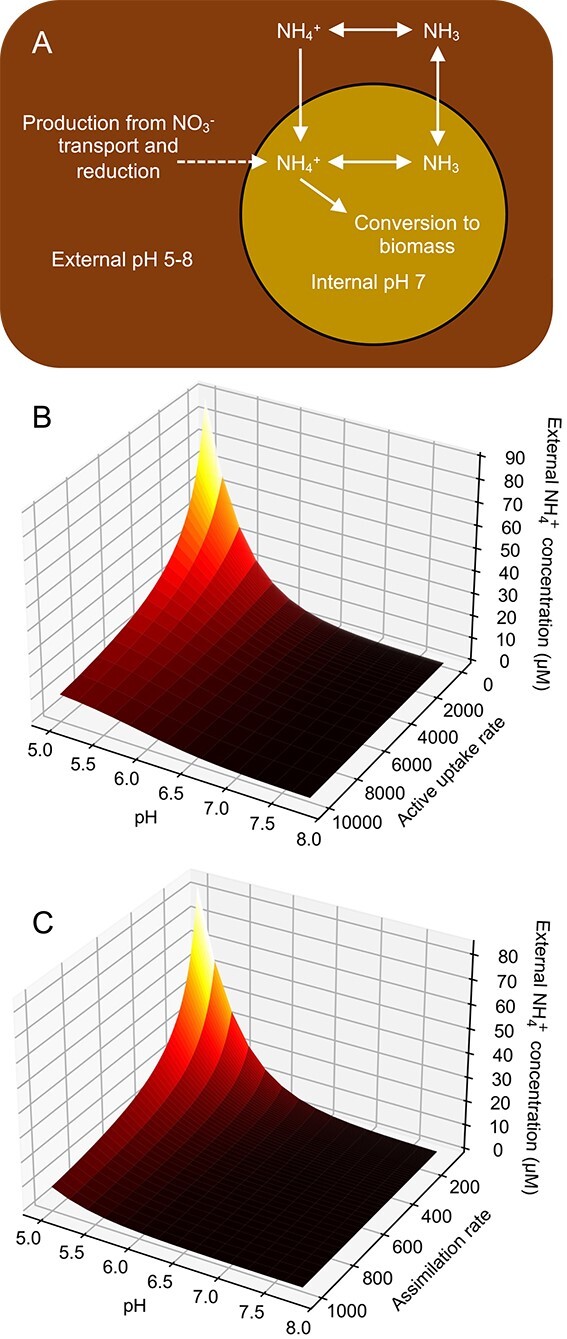
A) Diagram representing flow of N between compartments considered in our model and the pH values considered for each compartment. B&C) model simulations depicting the external NH^+^_4_ concentration given various pH and B) active uptake rates or C) assimilation rates for a single cell with a constant rate of internal NH^+^_4_ production.

To contextualise the above experimental results, we explored a simplified cellular growth model involving nitrate assimilation and ammonia leakage from cells, building on from a previously published model [37]. We considered a given internal ammonium production rate, mimicking nitrate uptake and subsequent reduction (Fig. 4A). We explored the effect of key parameters: ammonium uptake rate, ammonium assimilation rate (i.e. biomass incorporation rate) and environmental pH level. We found that increasing uptake or assimilation rates reduced loss of ammonia to the media and vice versa (Fig. 4B&C), although a certain level of leakage is always present even at high uptake and assimilation rates (Fig. 4B&C). Additionally, the impact of lowering the uptake or assimilation rate increases with lower environmental pH (Fig. 4B&C). This suggests that organisms must maintain high ammonium uptake or assimilation to negate the effects of ammonia leakage, particularly under low pH conditions. It may also suggest a benefit for” ammonium-scavenging” organisms to reduce their external pH, inducing leakage in others. This is especially pertinent in light of the observation that *S. indica* can reduce its external local pH (Fig. S11).

### Reduced ammonia uptake in *B. Subtilis* results in higher pH impact on its growth and increases its ability to support *S. Indica* growth in nitrate media

**Figure 5:**
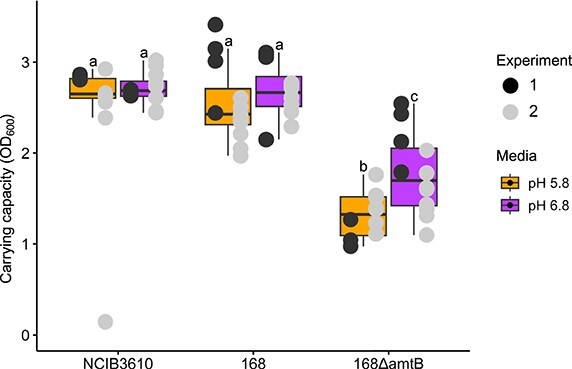
Carrying capacity of fitted bacterial growth curves for wild type *B. Subtilis* strains (NCIB3610 and 168) and the ammonium transporter mutant 168∆amtB in media with two levels of phosphate buffer composition (pH 5.8 and pH 6.8). Growth curves were measured in a plate reader every 30 mins, over the span of 94 hours incubated at 30°C and continually shaken at 200 rpm. Experiment was repeated twice, mid point indicates median, edges of boxes indicate lower (LQ) and upper quartiles (UQ) and ends of whiskers indicate maxima and minima excluding outliers, defined as points outside the bounds LQ/UQ −/+ 1.5·IQR (inter-quartile range). Individual well measurements are shown with points overlayed on the boxplots. Lettering above boxes indicates significant differences (pairwise Wilcoxon *P <* 0.05).

One key consideration arising from our model is that the reduction of active uptake of ammonium by a given cell would increase its leakage of ammonia to the environment, and that this effect would be exacerbated under lower pH. To test this theoretical finding, we used an ammonium uptake mutant (*B. subtilis* 168∆amtB) and assessed its ability to grow under different pH environments (Fig. 5). These experiments were performed in liquid culture, i.e. a well-mixed homogenous environment, to mimic the situation we modelled (Fig. 4A). Growth of wild-type *B. subtilis* was not affected significantly by a pH change from 6.8 to 5.8, whereas the mutant strain, with reduced active uptake of ammonium, displays significantly lower growth under reduced pH (Fig. 5). This supports the theoretical prediction that cells with reduced ammonium uptake will have a pH dependent impact on their ability to keep ammonium and therefore might be affected in their growth rate.

We expect a *B. subtilis* ammonium uptake mutant to have higher ammonia leakage (at any pH level). To test this, we have used the wildtype and the mutant strains to initiate co-cultures with *S. indica* on agar plates - we could not use supernatant-based liquid culture experiments, as this *B.subtilis* strain is a tryptophan auxotroph and added amino acid in monoculture would interfere with the ammonium-based interaction. On agar media with only nitrate as nitrogen source (including no tryptophan), we found that both the wildtype and the mutant can enhance *S. indica* growth, but the mutant can do so to a higher degree (Fig. S12). This finding supports the idea that the mutant with reduced ammonia uptake results in higher ammonia levels in its environment.

## Discussion

We have studied here the possibility of nitrogen sharing, among two common, plant-growth promoting microorganisms, *S. indica* and *B. subtilis*. This specific fungi-bacteria pair is previously shown to present a thiamine-mediated auxotrophic interaction [15], and the fungi *S. indica* was shown to be incapable of nitrate assimilation [29]. We re-confirmed the latter proposition and showed that *S. indica* is indeed incapable of growth when nitrate is the sole nitrogen source. We found, however, that this incapacity is lifted, and growth significantly enhanced, in the presence of *B. subtilis*. We find that this effect is mediated through ammonia, which is leaked from *B. subtilis*. We find that these results can be rationalised by a mathematical model, incorporating known permeability of ammonia to cell membranes, active ammonium uptake and assimilation, and ammonia-ammonium equilibrium. Utilising this model, we predict that some level of ammonium leakage is inevitable for cells and increases under low environmental pH and reduced uptake and assimilation rates. Taken together, these results experimentally prove that nitrogen sharing among soil microorganisms is a feasible and specific interaction mechanism, and that ammonia-based interactions can be influenced by environmental pH around the microorganisms and their individual ammonia uptake and assimilation rates.

The presented findings are relevant in our understanding of nitrogen dynamics in soils. Nitrate is a major component of global soils [11, 30–33] and applied fertilisers making it an abundant source of nitrogen for many microorganisms and plants. Assimilating nitrate is an energetically expensive process, necessitating both active uptake of nitrate and its reduction to ammonia [39, 40, 44]. Ammonia, free amino acids and more complex organic nitrogen-containing compounds also exist in global soils [45–48]. Microorganisms and plants have been demonstrated to have to ability to take up not only nitrate and ammonium but also free amino acids and more complex compounds including peptides and proteins [30, 49–51].

The presented finding that ammonia sharing can be possible for microorganisms that do not utilise nitrate suggest that this mechanism can allow such microorganisms to cut an energetic corner in their search for nitrogen and rely on the exuded/ leaked compounds provided by other members of a soil community. This mechanism could then lead to loss of genetic capacity to assimilate nitrate. The mechanism we describe here is likely not unique to ammonium and ammonia. Many organisms, including microorganisms, produce a range of volatile organic compounds that can diffuse through the gas phase [52]. As such, these cannot be held by the producer and may act as a nutrient source for surrounding organisms. More specifically, any chemical species that exists in equilibrium between an ion (non-permeable to cell membranes) and a dissolved gas (highly permeable) may exhibit similar dynamics exactly like ammonia and ammonium. This is true for at least two other important growth elements, carbon and sulfur. Carbonate ions and sulfate ions exist in equilibria with dissolved gaseous carbon dioxide and sulfur dioxide [53, 54] respectively.

The model presented here shows that ammonia leaked from cells will relate to their uptake and biomass incorporation rates. Subject to exact values of these parameters, we see a wide range of leaked ammonia concentrations are attainable in the vicinity of cells (Fig. S13). Indeed, we found, testing 3 other bacteria, common in the soil, that they have all leaked ammonia into culture supernatant when grown in nitrate-media (Fig. S10). The high membrane permeability of ammonia means that all organisms are ill-fated to leak nitrogen in this form. Furthermore, a broad spectrum of organisms are known to be incapable of growth on nitrate as the sole nitrogen source or are missing the required genetic machinery [42, 55–58]. This fact, coupled with our results, indicates that this mechanism of nitrogen sharing may be widespread in microbial communities and alludes to the potential prevalence of the loss of such machinery.

We have also identified here a small amount of ammonium production in the *S. indica* cultures grown on nitrate media and without bacterial supernatant supplementation. This suggests that *S. indica* can use spore stored nitrogen sources, or recycling of amino acids, to achieve some growth in nitrate media and ammonia is leaked as a consequence. Deamination of nucleic and amino acids, in nitrogen free media, has been observed in response to nitrogen limitation in *Escherichia coli* [59] and *Neurospora crassa* [60] respectively. Considering that this process of spore-stored nitrogen recycling and ammonia loss would be happening in tandem with ammonia assimilation from *B. subtilis* supernatant, we conclude that growth of *S. indica* in media supplemented with *B. subtilis* supernatant results in net ammonia consumption.

Ammonia dynamics can be influenced by environmental pH. Specifically, any nitrogen assimilating microbe will suffer at low pH and any impact of reduced assimilation will be exacerbated. Furthermore, any microbe relying on such leaked ammonia would benefit from lower pH in its local environment, provided sufficiently high ammonia uptake. Experimental results presented here align with these considerations, we found an ammonium transporter mutant of *B. subtilis* to be more susceptible to reduced growth in face of environmental pH reduction, and that *S. indica* promotes a low pH local environment upon growth. Additionally, *B. subtilis* may represent a particularly good” leaker” of ammonia; *B. subtilis GDH* may exclusively function to degrade, rather than produce, glutamate owing to a very low affinity for the ammonium ion [61, 62]. This would mean that in *B. subtilis* ammonia is only assimilated via *GS*. This would effectively reduce the assimilation rate described by our model and lead to higher rates of leakage into exterior media. These results also have implications in the context of soil. Low pH around *Arabidopsis* root systems has been shown to be vital in the suppression of plant immune responses by *Pseudomonas* [63]. Beneficial microorganisms also have to suppress plant immune responses to colonise host tissue [64, 65]. Thus, it is possible that pH lowering is a strategy used by *S. indica*, or other fungi, initially to aid in host immune responses to allow tissue colonisation and has subsequently allowed *S. indica* to utilise low pH environments and high ammonium uptake as an ammonium-scavenging strategy.

## Supplementary Material

Richards_etal_final_SI_wrae171

richards_etal_SupplementaryFigures_wrae171

richards_etal_DatafileS1_wrae171

## Data Availability

Raw data, where not fully presented in figures is available in Supplementary Data File S1. Python code used to run mathematical modelling is available on github at https://github.com/lukeZrich/RichardsNsharing2024
