# Supplementary material for: Ammonia leakage can underpin nitrogen-sharing among soil microorganisms
Source: ISME J. 2024 Sep 5;18(1):wrae171. doi: 10.1093/ismejo/wrae171 (PMC11440039; doi:10.1093/ismejo/wrae171)
Supplement: Richards_etal_final_SI_wrae171 [file richards_etal_final_si_wrae171.docx]

**Ammonia leakage can underpin nitrogen-sharing among soil microorganisms.**

Luke Richards[^1^](https://orcid.org/0000-0002-6449-9555), Kelsey Cremin^1^, Mary Coates^1^, Finley Vigor^1^, Patrick Schäfer[^2^](https://orcid.org/0000-0002-0366-6858), and Orkun S Soyer ^*,1^

# Supplementary Figures


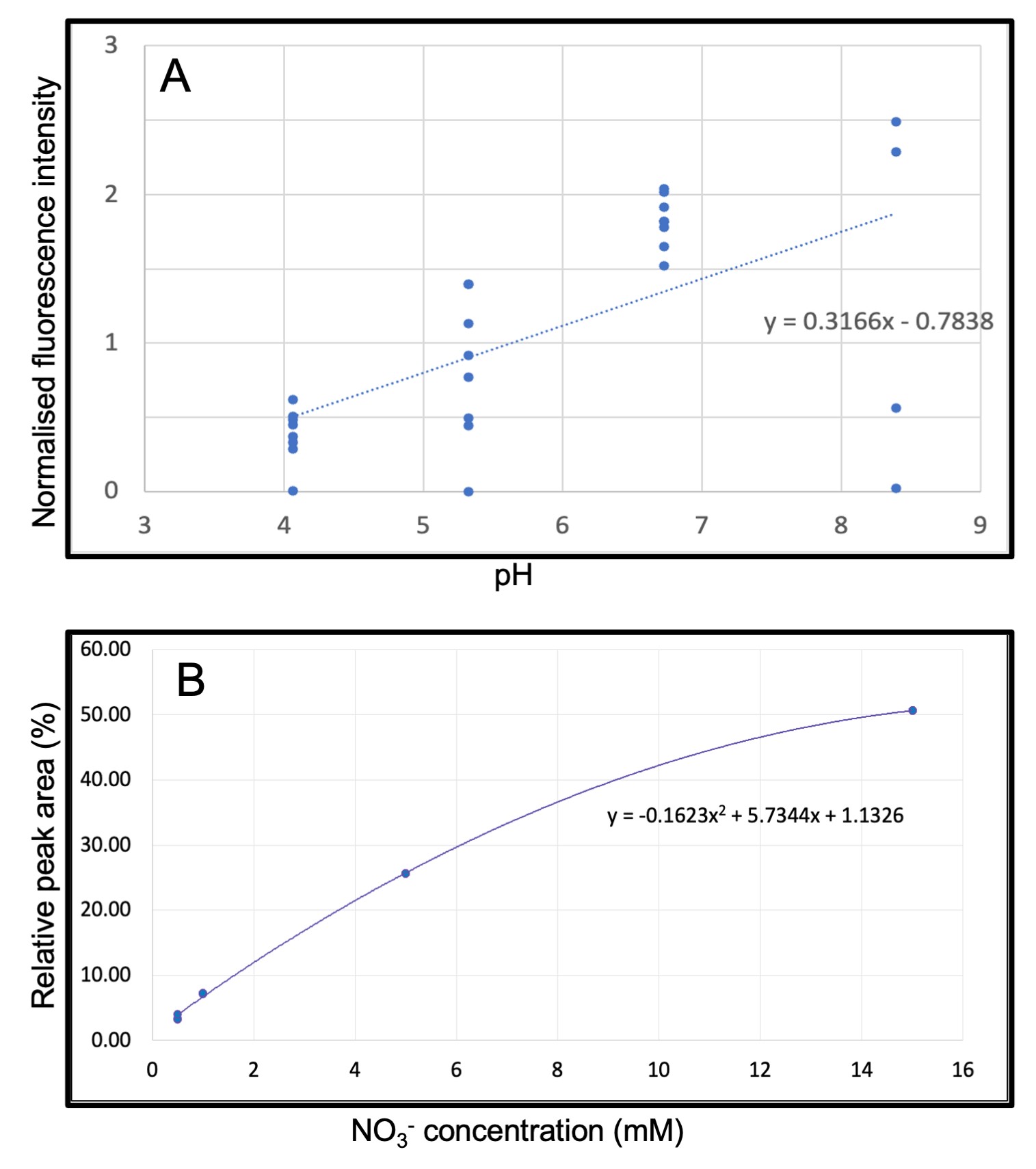


**Figure S1:** Standard curves and associated equations used for the quantification of A) pH, *R*^2^ = 0.38 and B) nitrate, *R*^2^ = 0.97.


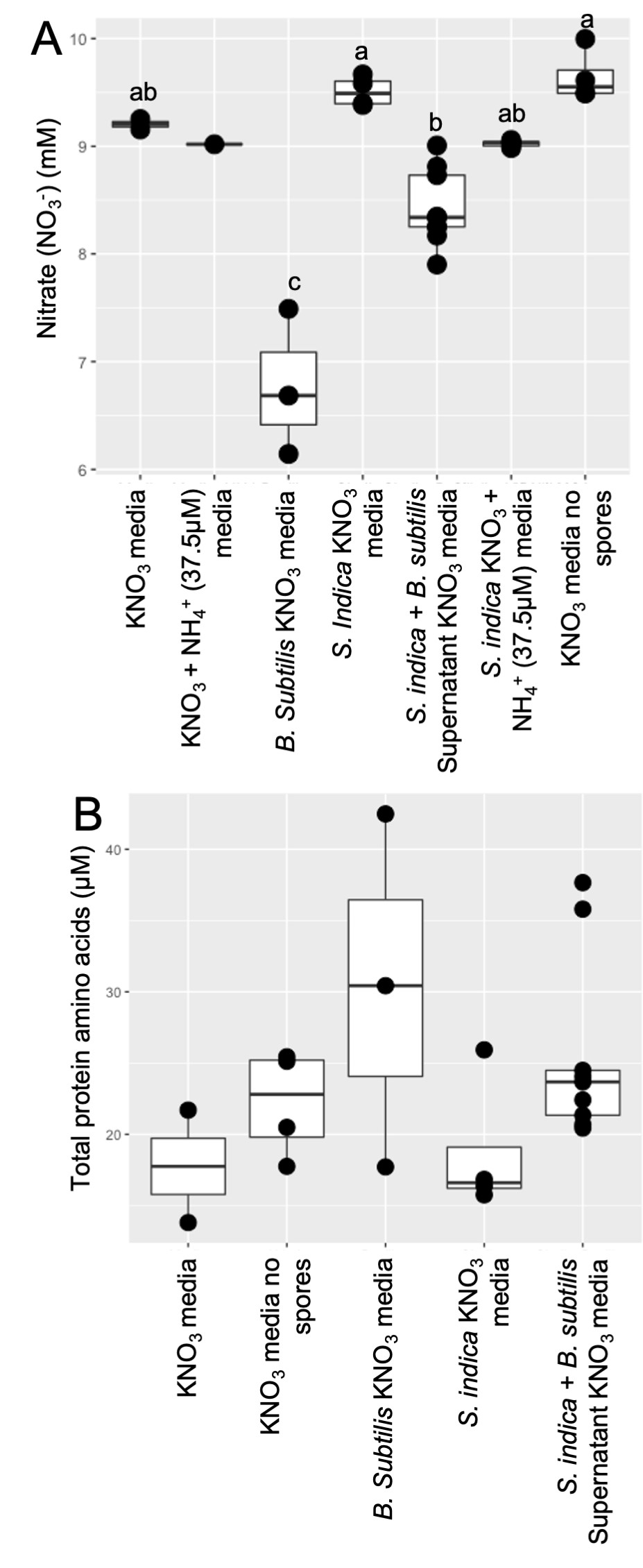


**Figure S2:** A) Nitrate and B) total protein-derived (after hydrolysis) amino acid quantification for *S. indica* and *B. subtilis* growth supernatants in various media and media only controls as indicated on the x axes. A) Significant differences are indicated with letters above bars (TukeyHSD p*<*0.05) (KNO
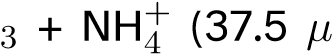
M) media was excluded from statistical analysis because this represents only one measurement). B) No statistical differences were found between any of the treatments (TukeyHSD *P <* 0.05). For both figures, mid point indicates median, edges of boxes indicate lower (LQ) and upper quartiles (UQ) and ends of whiskers indicate maxima and minima excluding outliers, defined as points outside the bounds LQ/UQ -/+ 1.5·IQR (Inter-quartile range)


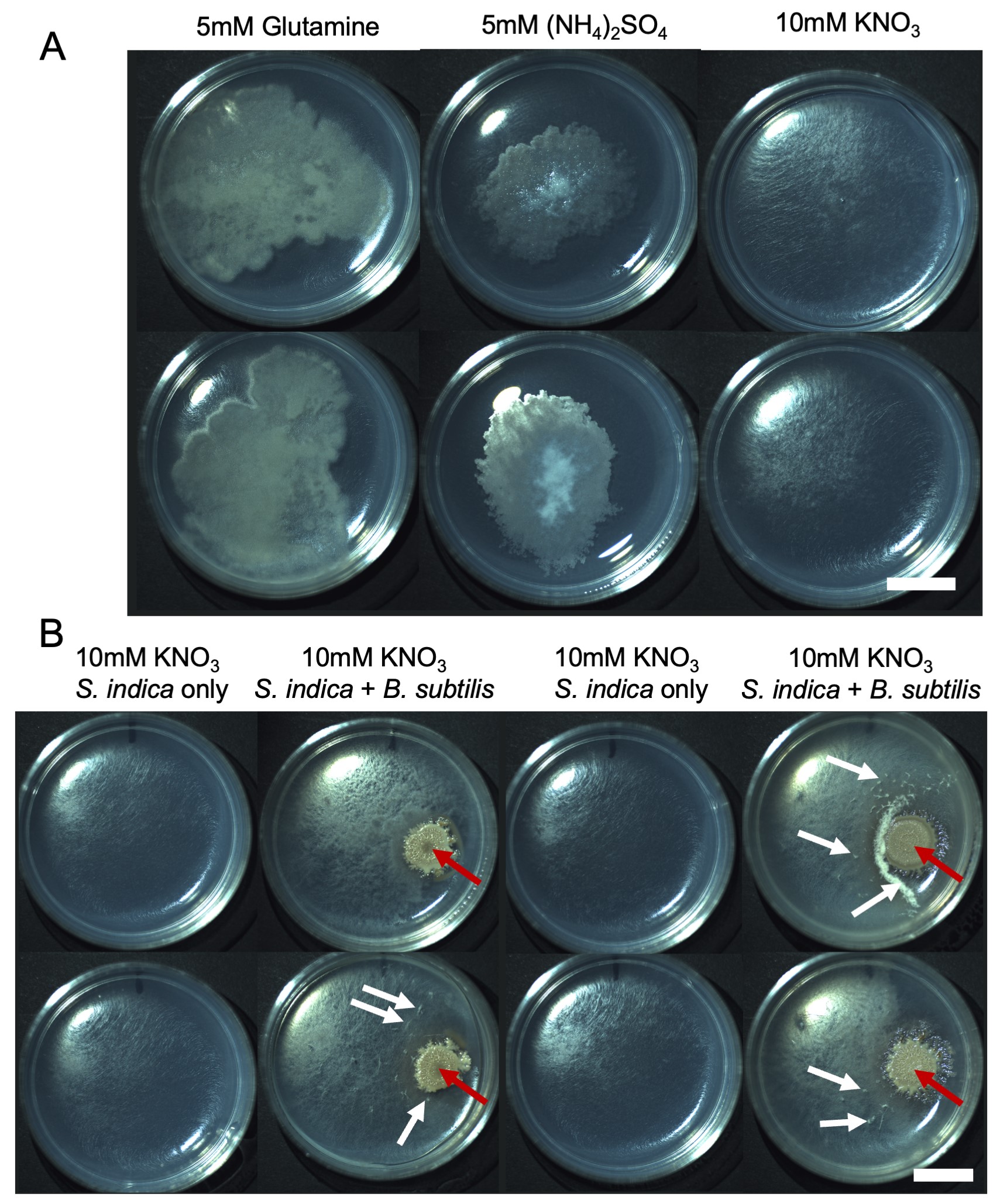


**Figure S3:** Additional replicates of A) *S. indica* growth after 42 days on ATS media supplemented with the indicated N-source. B) *S. indica* growth after 42 days in isolation or in the presence of *B. subtilis* on ATS media supplemented with 10 mM KNO_3_. *B. subtilis* inoculum was added 2 days after *S. indica* inoculation. On co-culture plates, large *B. subtilis* colonies are visible on the right, indicated with red arrows. In co-culture the mat of *S. indica* mycelia appears generally more dense but is also accompanied by ”fluffy” protrusions from the media, indicated with white arrows. The two sets of images shown on the left and right panels are from replicate experiments. Scale bar is 1 cm and applies to all panels.


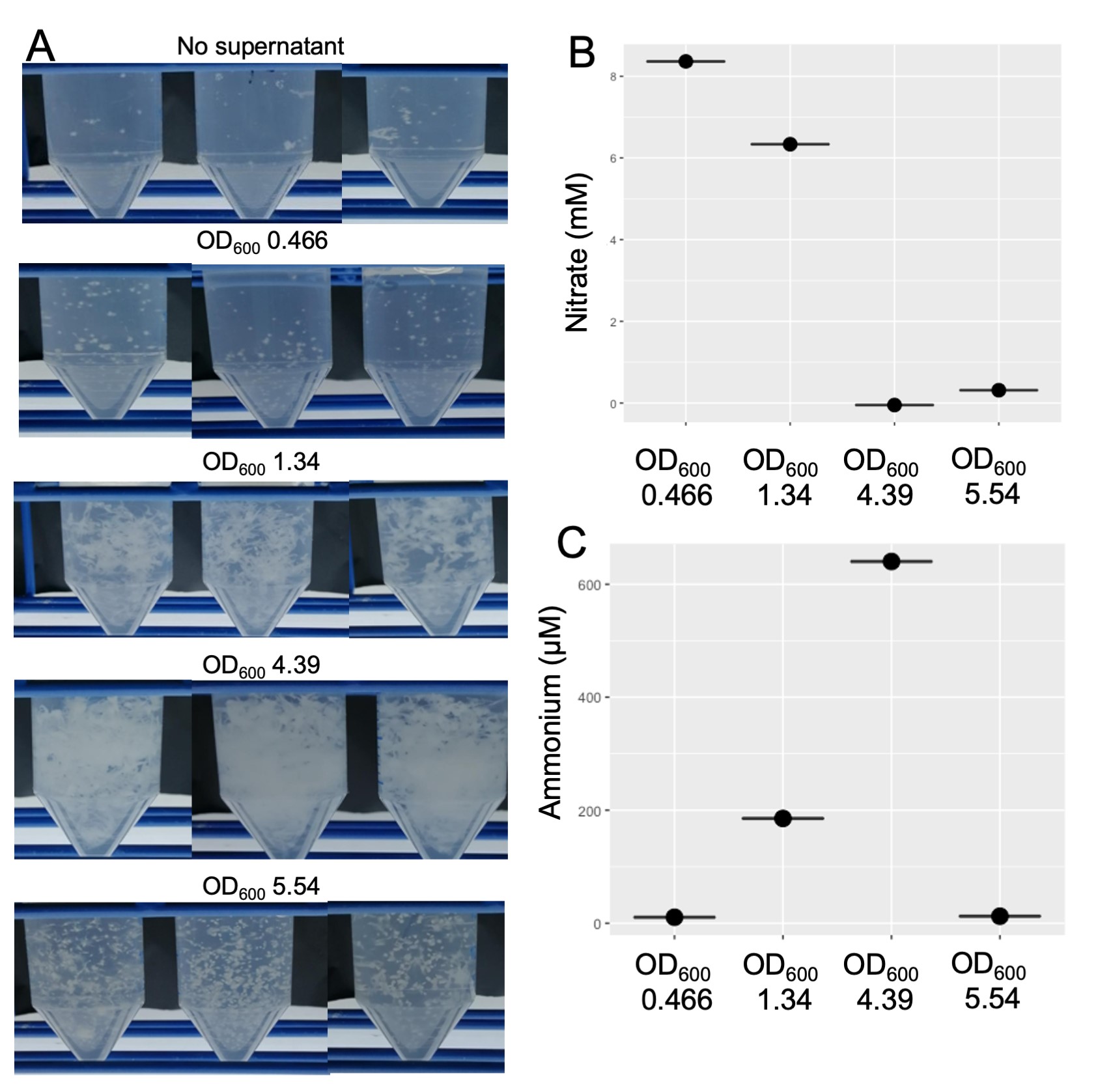


**Figure S4:** *B. subtilis* growth stage impacts *S. indica* growth]A) *S. indica* growth after 1 week of liquid culture in 50:50 mixtures of ATS 10 mM KNO_3_ and *B. subtilis* supernatants grown to different optical densities in the same media. B) Nitrate and C) ammonium quantification for the same *B. subtilis* supernatants used in A.


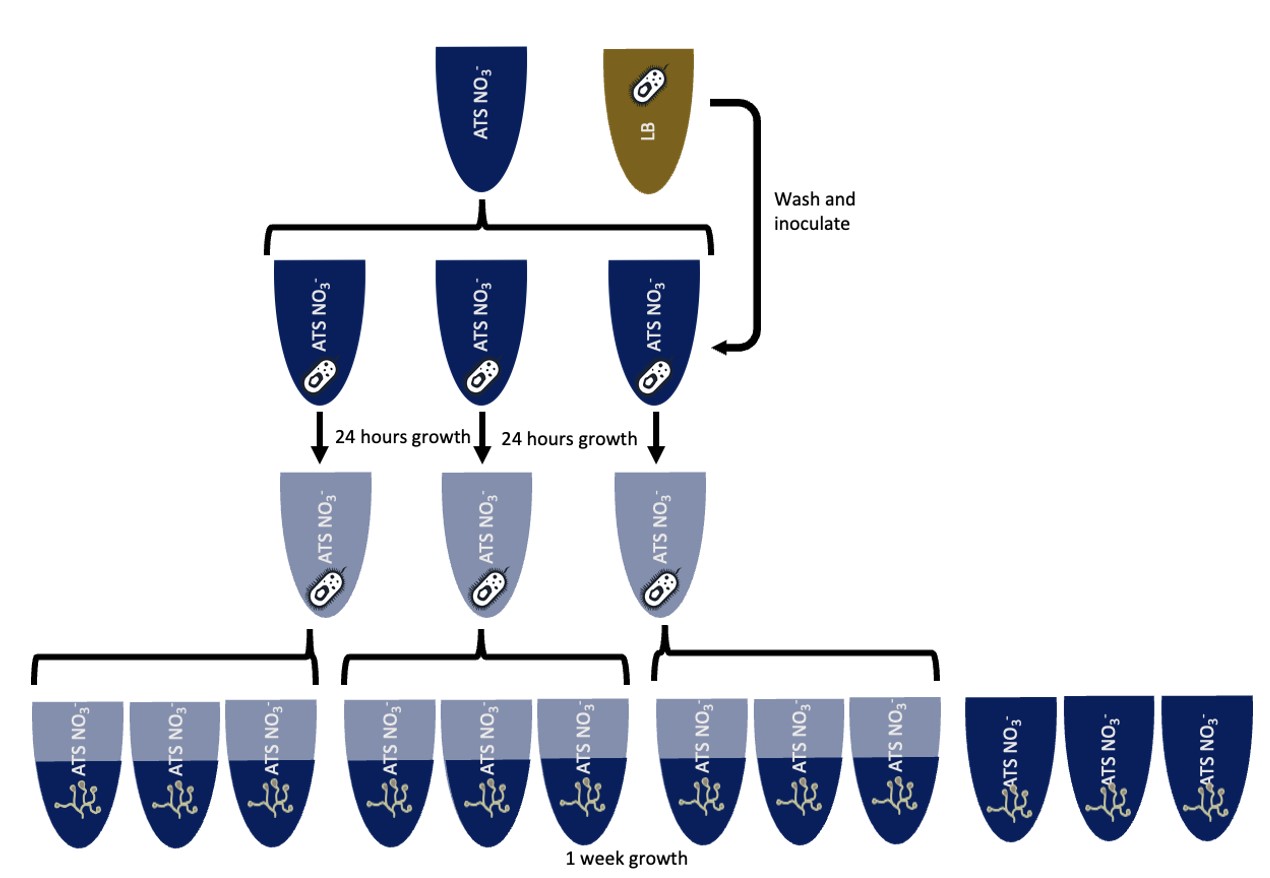


**Figure S5:** Experimental schematic to explain the basic experimental design for *S. indica* growth quantification in *B. subtilis* supernatants.


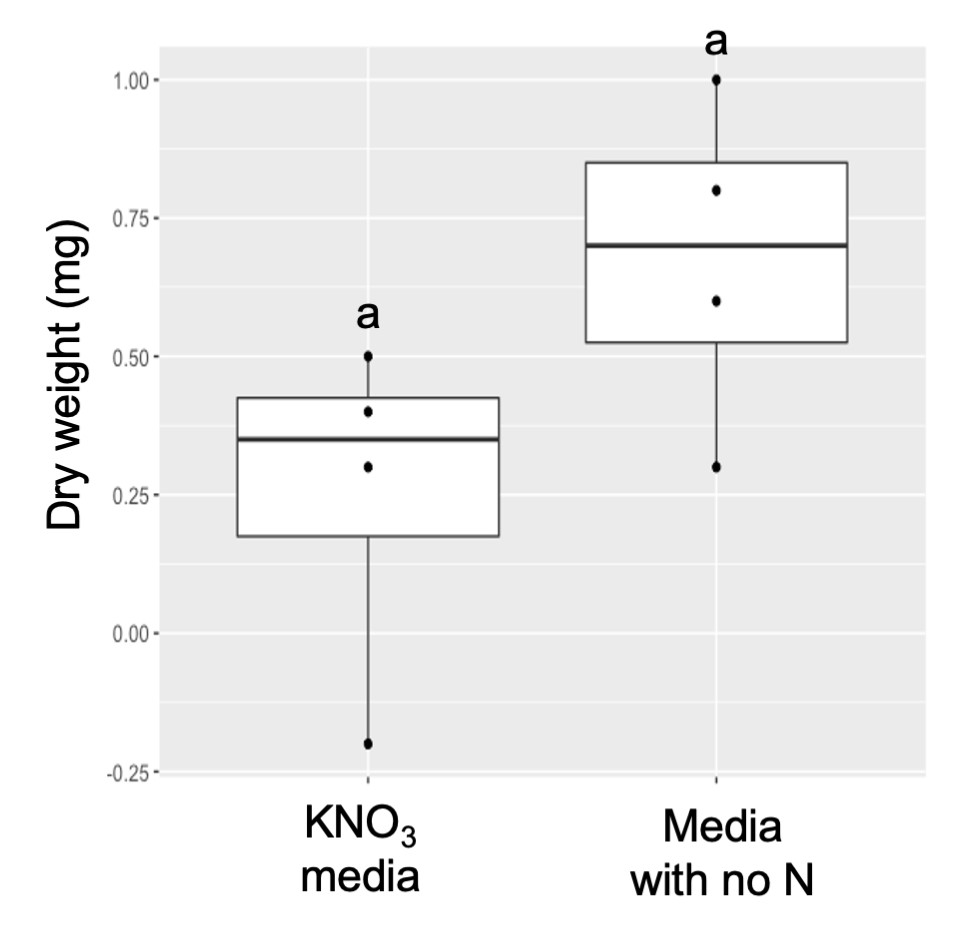


**Figure S6:** Dry weight of *S.indica* growth after 1 week of liquid culture in ATS media supplemented with 10 mM KNO_3_ or no N-source. Mid point indicates median, edges of boxes indicate lower (LQ) and upper quartiles (UQ) and ends of whiskers indicate maxima and minima excluding outliers, defined as points outside the bounds LQ/UQ -/+ 1.5·IQR (Inter-quartile range).


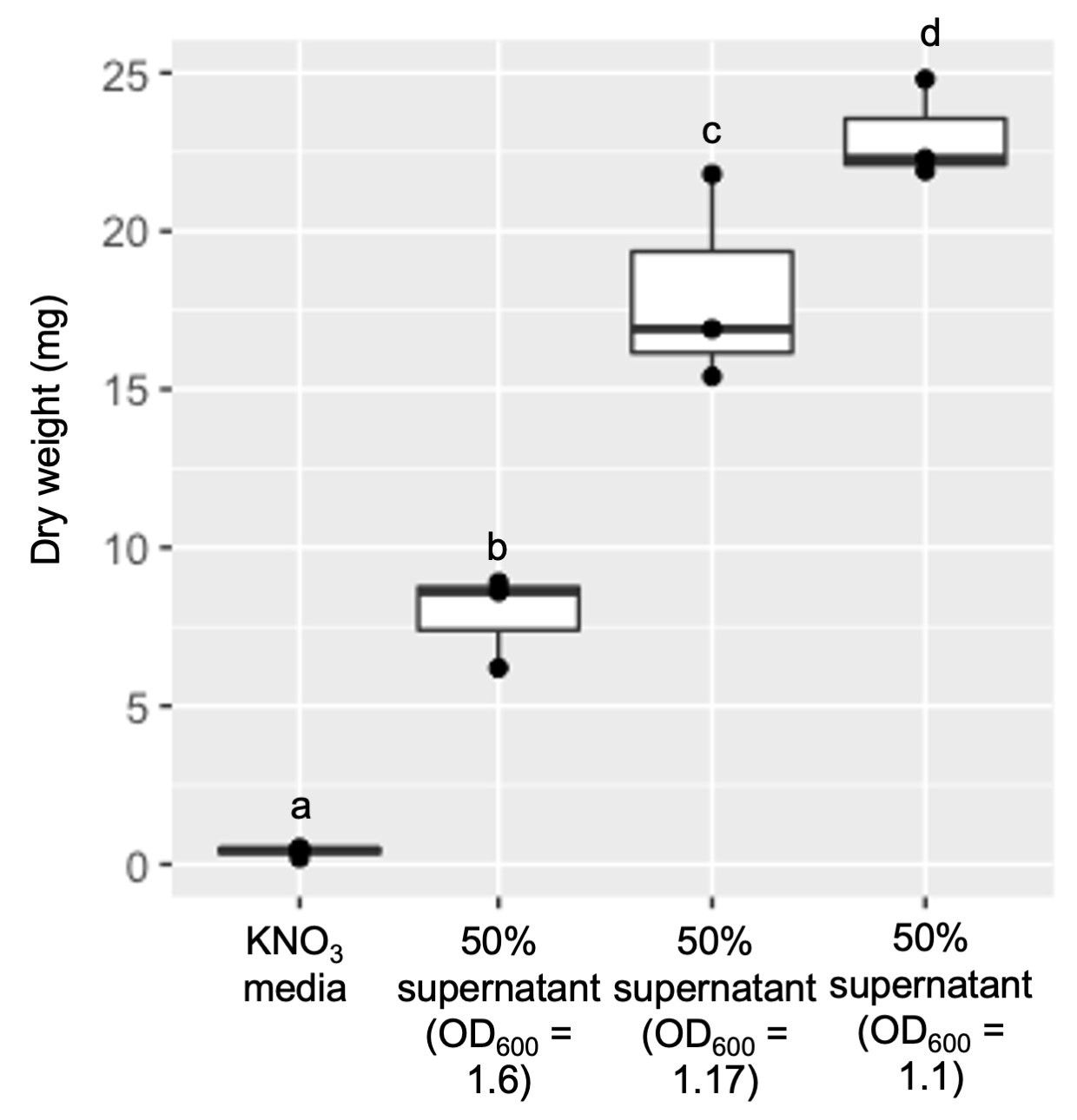


**Figure S7:** A) Dry weight of *S.indica* growth after 1 week of liquid culture in ATS media supplemented with 10 mM KNO_3_ or a 50:50 mixture of KNO_3_-only media and *B. subtilis* supernatant generated from KNO_3_-only media type to a bacterial optical density indicated on the x-axis. Mid point indicates median, edges of boxes indicate lower (LQ) and upper quartiles (UQ) and ends of whiskers indicate maxima and minima excluding outliers, defined as points outside the bounds LQ/UQ -/+ 1.5·IQR (Inter-quartile range). Significant differences are indicated by letters above bars (*P <* 0.05 Tukey HSD).


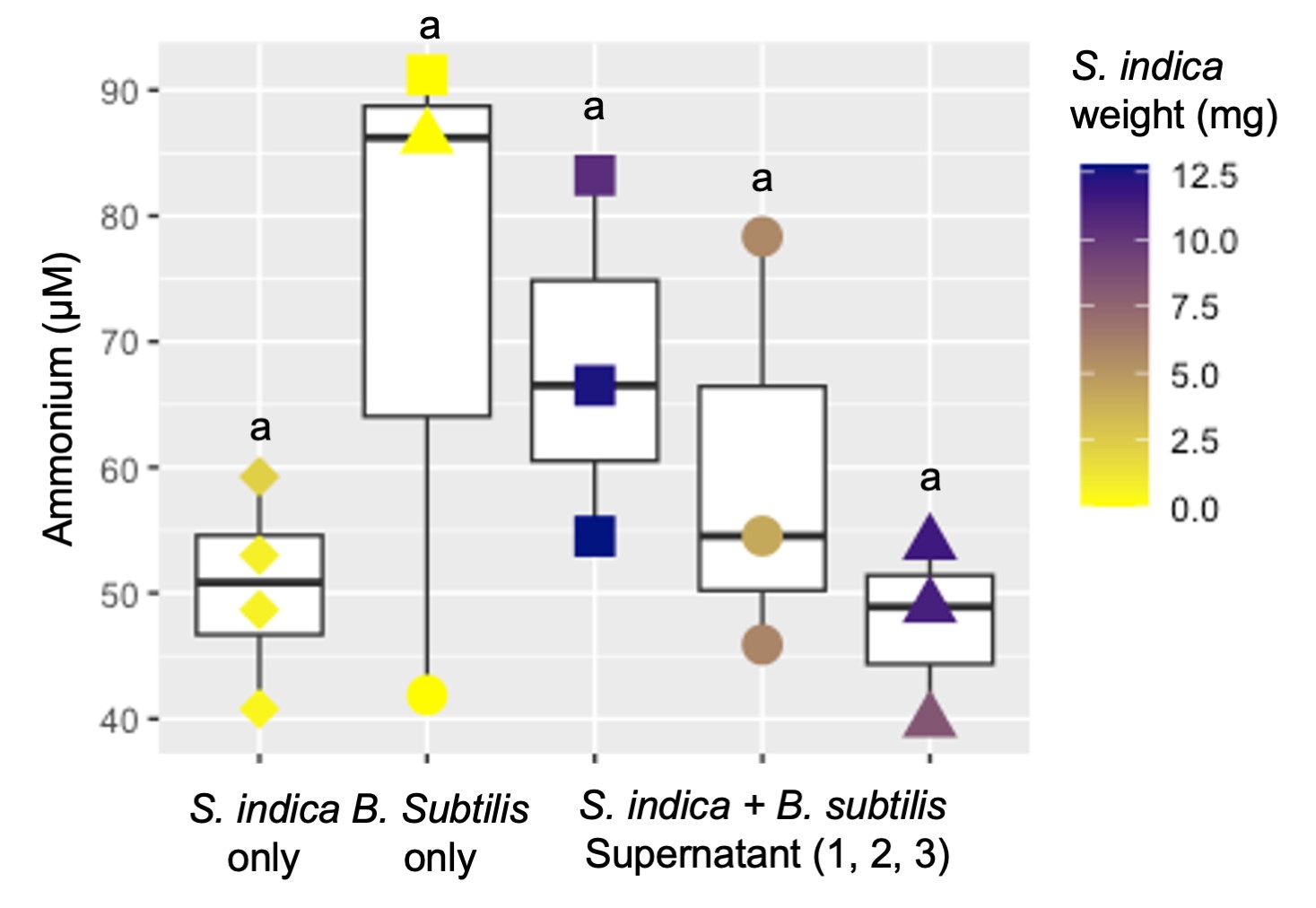


**Figure S8:** NH^+^_4_ concentration as measured by HPLC in *S. indica* and *B. subtilis* cultures in conditions indicated on the x-axis. Where applicable *S. indica* dry weight (from Figure 3A) for each individual point is indicated with a colour scale. Mid point indicates median, edges of boxes indicate lower (LQ) and upper quartiles (UQ) and ends of whiskers indicate maxima and minima excluding outliers, defined as points outside the bounds LQ/UQ -/+ 1.5·IQR (Interquartile range). Significant differences are indicated with letters above boxes (TukeyHSD *P <* 0.05). Plot point shapes indicate the OD_600_ of the *B. subtilis* culture at the time of supernatant harvest (from Figure 3A): diamond - NA, square - 1.02, circle - 1.83, triangle - 1.26.


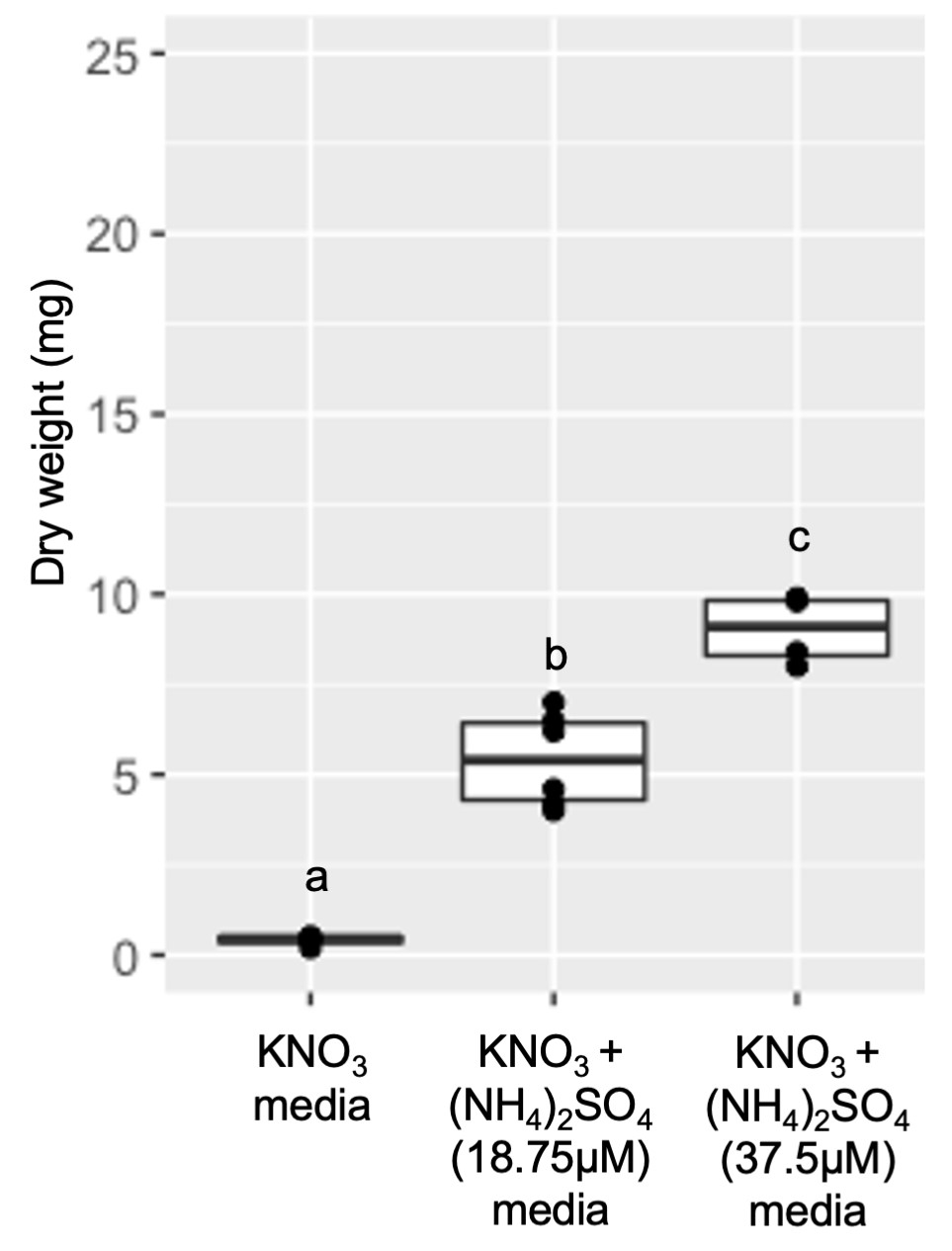


**Figure S9:** A) Dry weight of *S.indica* growth after 1 week of liquid culture in ATS media supplemented with 10 mM KNO_3_, 10 mM KNO_3_ + 18.75 *µ*M (NH_4_)_2_SO_4_ or 10 mM KNO_3_ + 37.5µM

(NH_4_)_2_SO_4_. Mid point indicates median, edges of boxes indicate lower (LQ) and upper quartiles (UQ) and ends of whiskers indicate maxima and minima excluding outliers, defined as points outside the bounds LQ/UQ -/+ 1.5·IQR (Inter-quartile range). Significant differences are indicated by letters above bars (*P <* 0.05 Tukey HSD).


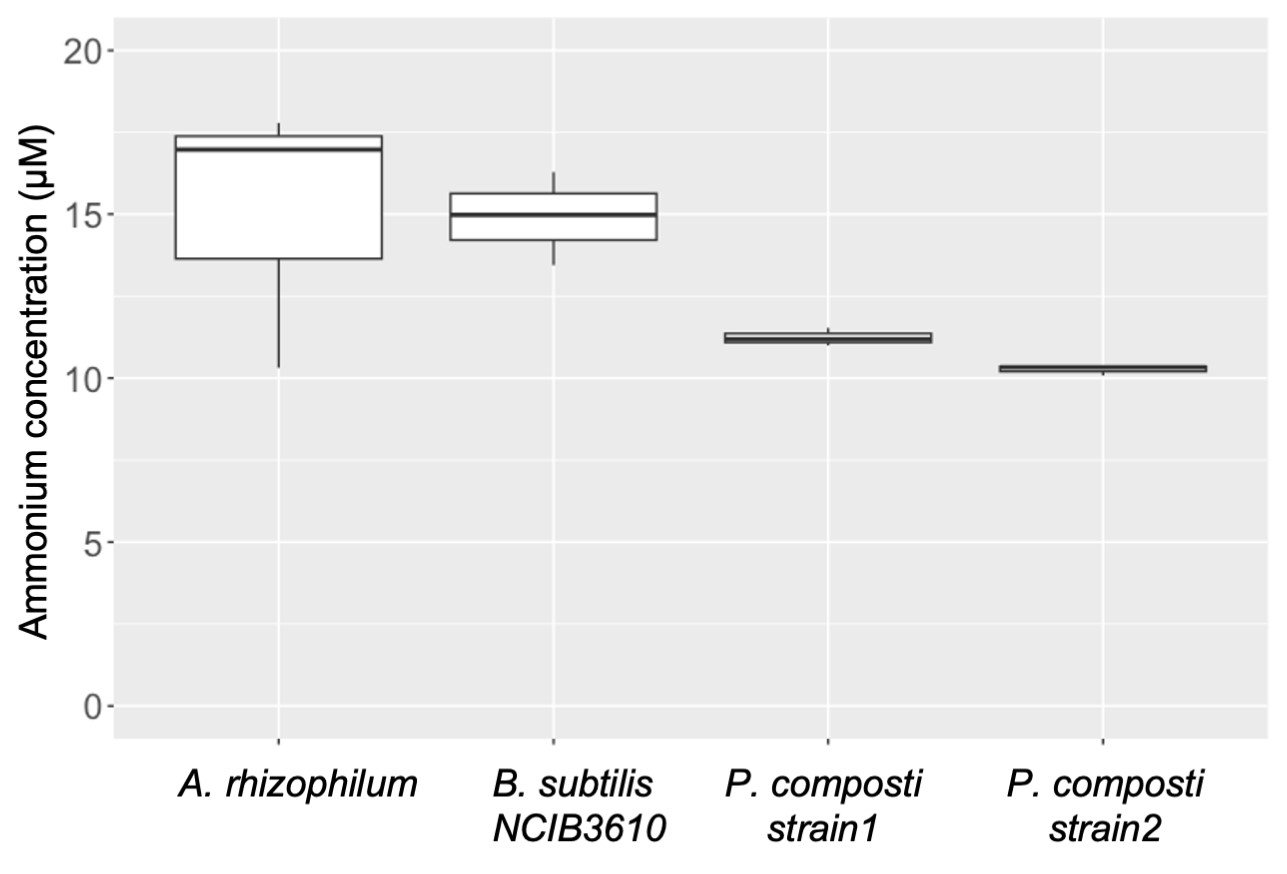


**Figure S10:** Supernatant ammonia concentration bacterial growth in BG11+ media (see Methods). *Allorhizobium rhizophilum* and two strains of *Pseudomonas composti* are strains recently isolated from an environmental sample (see Methods). *B. subtilis* is included as comparison. OD_600_ at sampling is indicated in supplementary data file S1. Mid point indicates median, edges of boxes indicate lower (LQ) and upper quartiles (UQ) and ends of whiskers indicate maxima and minima. No significant differences were identified between strains (*P <* 0.05 Tukey HSD).


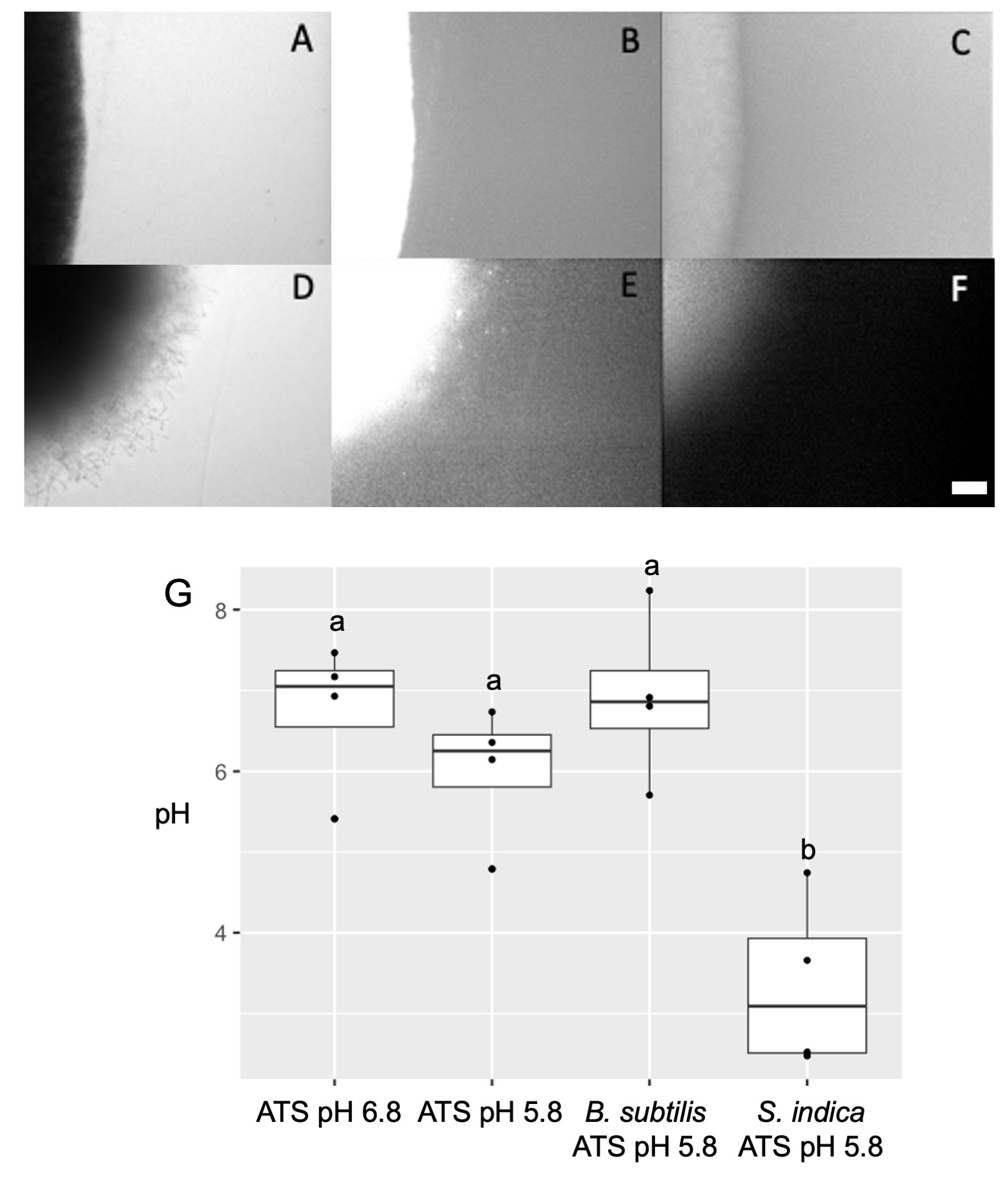


**Figure S11:** A and D) Bright field, B and E) RED fluorescence (PI) and, C and F) GFP fluorescence (BCECF pH sensitive) images of A, B and C) *B. subtilis* and, D, E and F) *S. indica* grown in ATS media pH5.8 and flooded with 10*µ*M BCECF pH sensitive dye and 100 *µ*M PI (propidium iodide). Scale bar indicates 100 *µ*m. G shows pH values calculated using BCECF fluorescence values normalised against RED fluorescence for two abiotic media plates at pH 5.8 and 6.8, and *B. subtilis* and *S. indica* plates grown at pH 5.8. Fluorescence images for pH calculations were taken in an are directly adjacent to grown colonies but without colonies in view. Mid point indicates median, edges of boxes indicate lower (LQ) and upper quartiles (UQ) and ends of whiskers indicate maxima and minima excluding outliers, defined as points outside the bounds LQ/UQ -/+ 1.5·IQR (Inter-quartile range). Significant differences are indicated by letters above bars (*P <* 0.05 Tukey HSD)


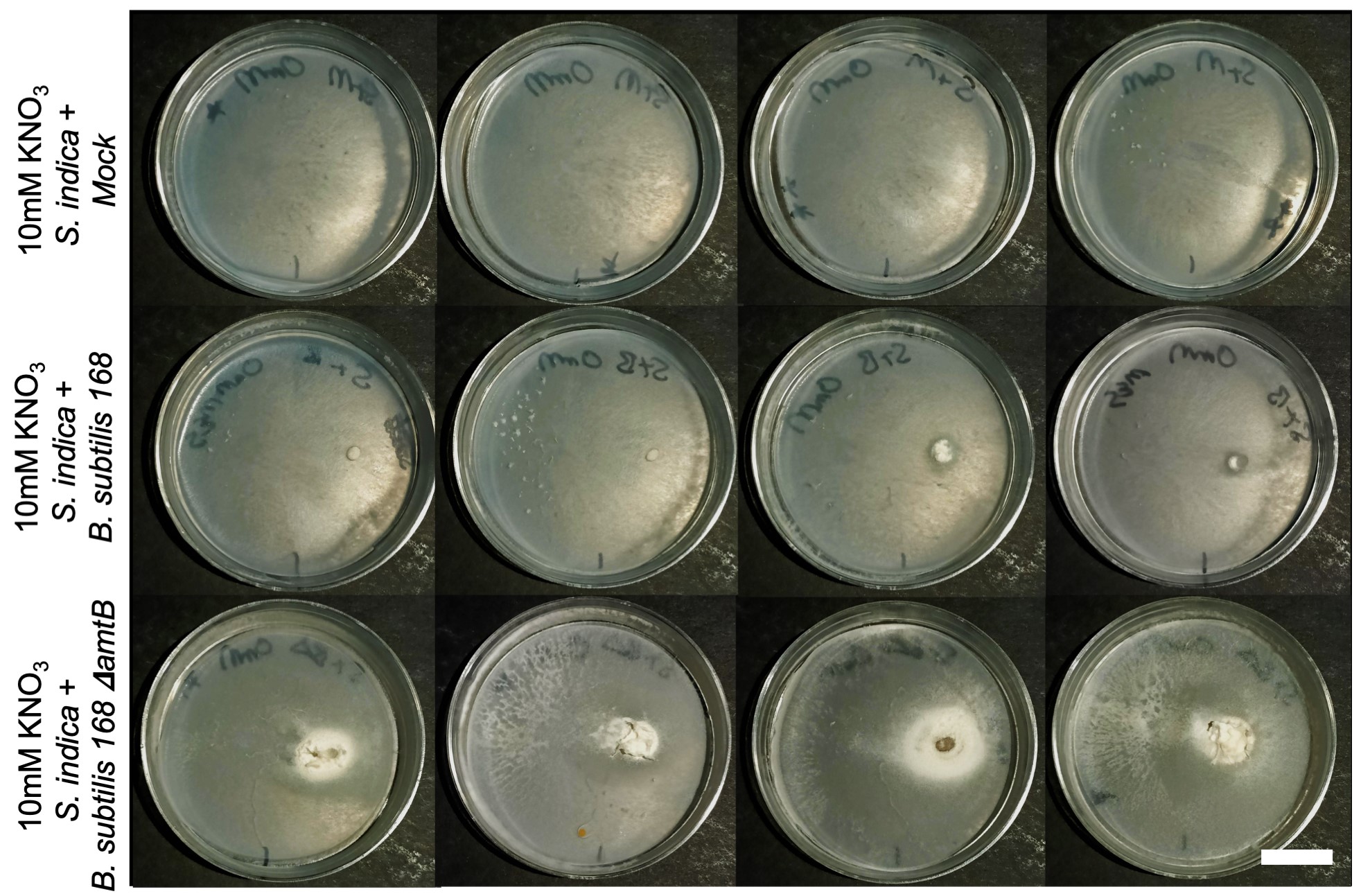


**Figure S12:** *S. indica* grown in isolation or in the presence of *B. subtilis* 168 or the ammonium uptake mutant on ATS media supplemented with 10 mM KNO_3_ for 22 days. *B. subtilis* inoculum was added on the RHS of plates 2 days after *S. indica* inoculation on the LHS. On co-culture plates *B. subtilis* colonies are not visible at the site of inoculation. In co-culture the *S. indica* mycelia appears more dense at the site of inoculation. Scale bar is 1cm.


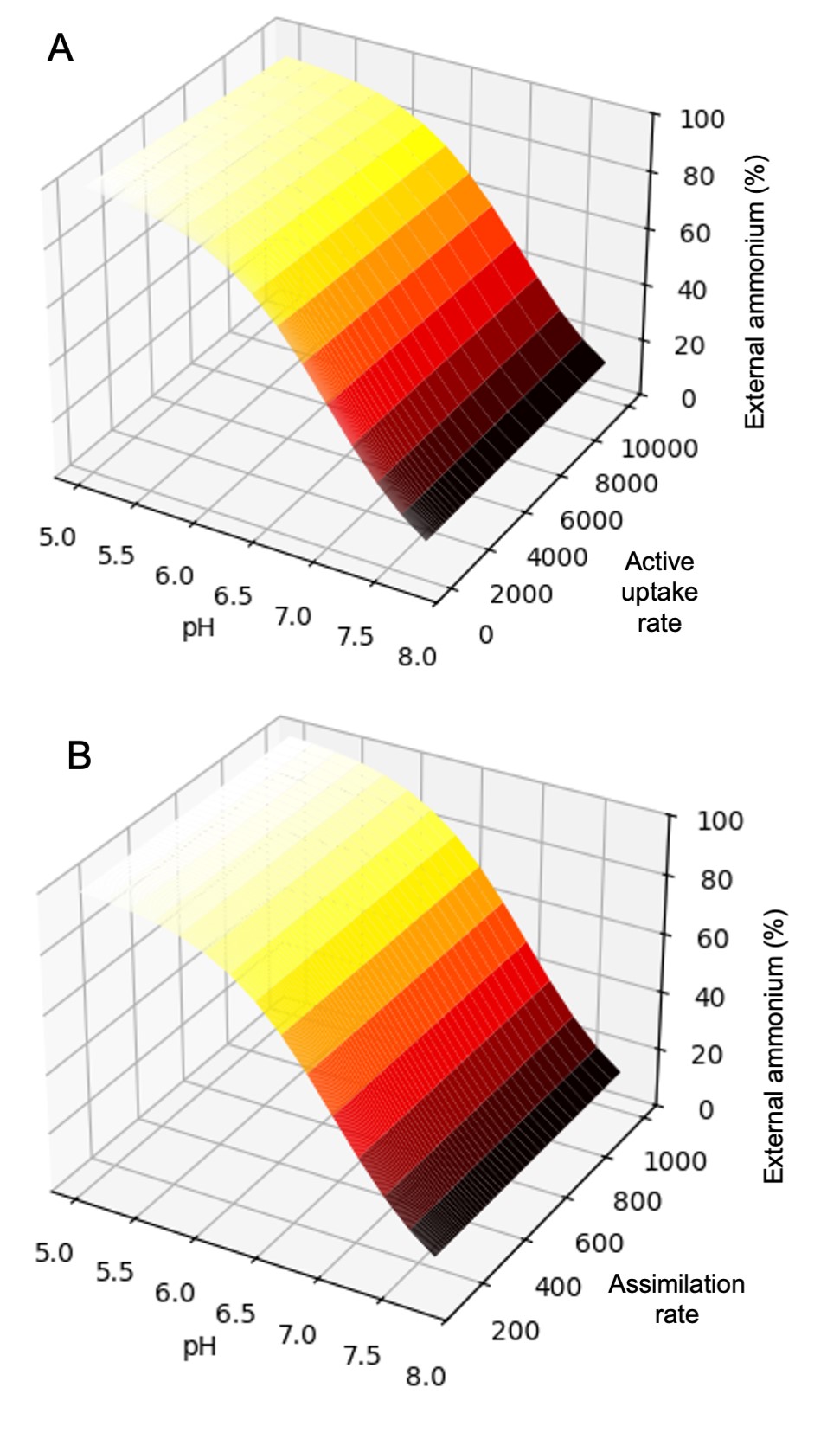


**Figure S13:** Model simulations depicting the percentage of free NH^+^_4_ that is outside the cell in our simulations given various pH and A) Active uptake rates or B) Assimilation rates for a single cell with a constant rate of internal NH^+^_4_ production.
